# Heavy Metal Tolerance in *Stenotrophomonas maltophilia*

**DOI:** 10.1371/journal.pone.0001539

**Published:** 2008-02-06

**Authors:** Delphine Pages, Jerome Rose, Sandrine Conrod, Stephane Cuine, Patrick Carrier, Thierry Heulin, Wafa Achouak

**Affiliations:** 1 Commissariat à l'Energie Atomique (CEA), Cadarache, Direction des Sciences du Vivant (DSV), Institut de Biologie Environnementale et Biotechnologie (IBEB), Lab Ecol Microb Rhizosphere & Environ Extrem (LEMiRE), Saint-Paul-lez-Durance, France; 2 Centre National de la Recherche Scientifique (CNRS), UMR Biol Veget & Microbiol Enviro, Saint-Paul-lez-Durance, France; 3 Aix-Marseille Université, Saint-Paul-lez-Durance, France; 4 Centre Européen de Recherche et d'Enseignement des Géosciences de l'Environnement (CEREGE), UMR 6635 CNRS- Universite Aix Marseille, IFR 112 PMSE Europole de l'Arbois, Aix en Provence, France; 5 Commissariat à l'Energie Atomique (CEA), Cadarache, Direction des Sciences du Vivant (DSV), Institut de Biologie Environnementale et Biotechnologie (IBEB), Service de Biologie Végétale et de Microbiologie Environnementale (SBVME), Lab Bioenerget Biotechnol Bacteries & Microalgues (LB3M), Saint-Paul-lez-Durance, France; University of Wyoming, United States of America

## Abstract

*Stenotrophomonas maltophilia* is an aerobic, non-fermentative Gram-negative bacterium widespread in the environment. *S. maltophilia* Sm777 exhibits innate resistance to multiple antimicrobial agents. Furthermore, this bacterium tolerates high levels (0.1 to 50 mM) of various toxic metals, such as Cd, Pb, Co, Zn, Hg, Ag, selenite, tellurite and uranyl. *S. maltophilia* Sm777 was able to grow in the presence of 50 mM selenite and 25 mM tellurite and to reduce them to elemental selenium (Se^0^) and tellurium (Te^0^) respectively. Transmission electron microscopy and energy dispersive X-ray analysis showed cytoplasmic nanometer-sized electron-dense Se^0^ granules and Te^0^ crystals. Moreover, this bacterium can withstand up to 2 mM CdCl_2_ and accumulate this metal up to 4% of its biomass. The analysis of soluble thiols in response to ten different metals showed eightfold increase of the intracellular pool of cysteine only in response to cadmium. Measurements by Cd K-edge EXAFS spectroscopy indicated the formation of Cd-S clusters in strain Sm777. Cysteine is likely to be involved in Cd tolerance and in CdS-clusters formation. Our data suggest that besides high tolerance to antibiotics by efflux mechanisms, *S. maltophilia* Sm777 has developed at least two different mechanisms to overcome metal toxicity, reduction of oxyanions to non-toxic elemental ions and detoxification of Cd into CdS.

## Introduction

*Stenotrophomonas maltophilia* is an aerobic, non-fermentative Gram-negative bacterium widespread in the environment. This species constitutes one of the dominant rhizosphere inhabitant, frequently isolated from the rhizosphere of wheat, oat, cucumber, maize, oilseed rape, and potato [1]–[4]. *S. maltophilia* shows plant growth-promoting activity as well as antagonistic properties against plant pathogens. It is currently being studied for its biological control of plant pathogens and was therefore utilized for the development of biopesticides [5]. *S. maltophilia* is also able to degrade xenobiotic compounds [6], [7], to detoxify high molecular weight polycyclic aromatic hydrocarbons [8], possessing therefore a potential for soil decontamination (bioremediation). This bacterium was also increasingly described as an important nosocomial pathogen in debilitated and immunodeficient patients [9], [10], as well as associated with a broad spectrum of clinical syndromes, e.g. bacteraemia, endocarditis, respiratory tract infections [11]. *S. maltophilia* displays intrinsic resistance to many antibiotics, making selection of optimal therapy difficult. The mechanisms underlying this multiresistance to drugs seem to result from a combination of reduced permeability [12], and expression of efflux pumps. Two RND efflux systems have been identified, SmeABC [13] and SmeDEF [14], [15].

Considering on one hand the rhizospheric origin of various opportunistic pathogens [16] including *S. maltophilia* and, on the other hand, the description of horizontal gene transfers in the rhizosphere [17], the tolerance of this bacterium to a wide range of toxic oxianions and metals must be addressed.

In the present study, we evidenced the tolerance of the strain Sm777 that belongs to *S. maltophilia* species, to very high concentrations of various toxic metals, especially cadmium, selenium and tellurium, involving two different tolerance mechanisms.

## Results and Discussion

The strain Sm777 was isolated as a culture contaminant associated to *Pseudomonas* strains and was revealed in a contest of heavy metal tolerance studies. This rod-shaped bacterium was persistent in cultures containing a high concentration of cadmium, and was identified as a *Stenotrophomonas maltophilia* by 16S rDNA sequencing. The sequence analysis (using the BLAST database of the National Center for Biotechnology Information; [http://www.ncbi.nlm.nih.gov]) showed that strain Sm777 matched 99.5% with 16S rDNA of the *S. maltophilia* LMG 958^T^ (accession n° DQ469587).

### MICs of drugs and heavy metals

*S. maltophilia* Sm777 was able to grow during 16 h in the presence of 500 µM CdCl_2_, 20 mM tellurite or 50 mM selenite without any significant increase of the lag phase. It is worthnoting that strain Sm777 also grew to a high density (10^9^ cfu.ml^−1^) in the presence of high concentrations of other heavy metals (0.1 mM CoCl_2_, 5 mM CuSO_4_, 4 mM ZnSO_4_, 10 mM NiSO_4_, 0.05 mM HgCl_2_, 0.02 mM AgNO_3_, >1 mM uranyl, and 5 mM Pb(NO_3_)_2_). Moreover, this bacterium was resistant to a wide range of antibiotics, such as kanamycin (50 µg.ml^−1^), gentamycin (100 µg.ml^−1^), tetracycline (50 µg.ml^−1^), and 50 µg.ml^−1^ of nalidixic acid. These may suggest that strain Sm777 overproduces some multidrug resistance (MDR) efflux pumps that are known to be involved in bacterial resistance to a wide range of compounds by extruding antibiotics and other toxic compounds.

### Oxianions reduction

To verify the hypothesis of overexpression of efflux systems to get ride of drugs and heavy metals, we analysed and localized the elemental composition of bacteria grown in the presence of tellurite and selenite, by using Energy Dispersive X-ray Spectroscopy (EDX) in conjunction with Transmission Electron Microscopy (TEM) or Environmental Scanning Electron Microscopy (ESEM).

**Figure 1:**
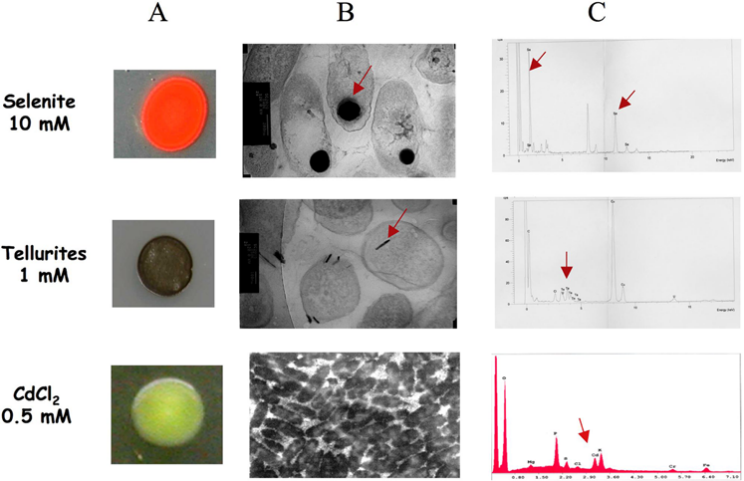
ESEM-EDX and TEM-EDX observations. Microscopic observations and representative energy-dispersive X-ray spectra of electron-dense particles of *S. maltophilia* Sm777 cells grown in ten fold-diluted TSB medium solidified with 15 g.l^−1^ agar, and supplemented with metals. (A & B) Colony shape, TEM-EDX micrographs and spectra of *S. maltophilia* Sm777 grown in the presence of selenite (10 mM) and tellurite (1 mM). (C) Colony shape, ESEM-EDX observation and analysis of cells grown in the presence of CdCl_2_ (500 µM). Arrows on micrographs indicate the presence of intracellularly localized electron-dense particles of Se and Te, and arrows on spectra indicate metal-specific peak detected.

The chemical microanalysis (TEM-EDX) of reddish colonies of strain Sm777 grown in the presence of selenite revealed cytoplasmic electron-dense Se^0^ granules (Fig. 1A). No detectable extracellular particles were observed. The intracellular Se^0^ granules strongly suggest that selenite tolerance of strain Sm777 is not related to an efficient efflux system. On the contrary, a *S. maltophilia* strain isolated from a seleniferous agricultural drainage pond sediment was shown to transform selenate and selenite and to form spherical extracellular deposits consisting of Se [18]. TEM-EDX observations of black colonies of strain Sm777 grown in the presence of tellurite revealed the presence of Te^0^ crystals in the cytoplasm and proved that tellurite was taken up by the cells and was reduced into tellurium in the intracellular compartment (Fig. 1B).

Active efflux of the metal is a frequently utilized strategy to produce tolerance by lowering the intracellular concentration to subtoxic levels. However, our data showing intracellular nanometer-sized particles of elemental selenium or tellurium, suggest that MDR efflux pumps probably do not mediate the heavy metal tolerance mechanism in strain Sm777 since tellurite and selenite-tolerance was associated to an intracellular reduction of these oxyanions and then by their accumulation.

### Tolerance of S. maltophilia to cadmium

ESEM observations coupled to EDX analysis of strain Sm777 grown in the presence of 500 µM CdCl_2_ revealed the presence of Cd associated to bacterial cells, but did not allow localizing it exactly (Fig. 1C). The bacterial Cd content was determined by ICP-AES as previously described [19]. This analysis revealed an accumulation of Cd strongly associated with the bacterial cell wall or incorporated into cells. Hence, this strain was able to accumulate Cd representing up to 4% of its dry mass. The presence of a cluster of genes from Gram-positive bacteria involved in both antibiotic and heavy metal resistance has been described in *S. maltophilia* D457R [20]. This cluster contains genes encoding a macrolide phosphotransferase (*mphBM*) and a cadmium efflux determinant (*cadA*). This study indicated a lateral gene transfer between Gram-positive and Gram-negative bacteria. The role of these genes in heavy metal tolerance of *S. maltophilia* has not been clearly evidenced yet.

### Cysteine accumulation in response to cadmium

**Figure 2:**
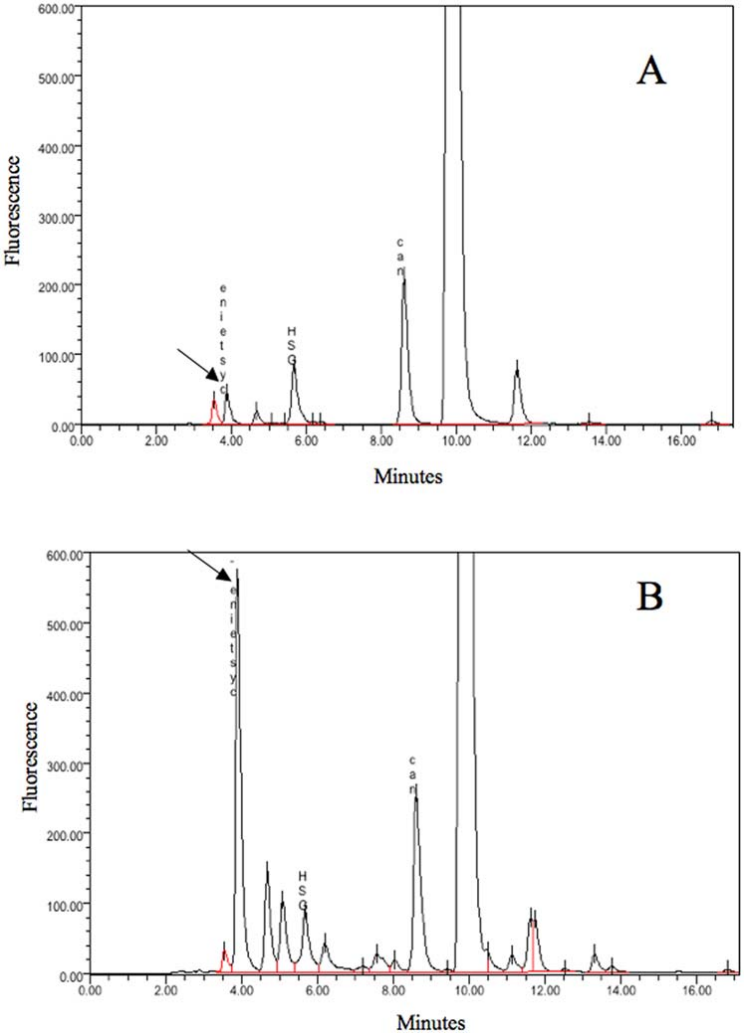
Soluble thiols analysis. HPLC analysis of nonprotein thiols in *S. maltophilia* Sm777 grown in TSB/10 without (A) or supplemented (B) with 500 µM of CdCl_2_. The arrow indicates cysteine peack. *N*-acetyl-L-cysteine (NAC) was used as an internal standard.

The role of thiol compounds in the protection against heavy metals is well known [21]. Moreover, the chemical sequestration of Cd is thought to occur by coordination of cysteine thiolate groups. For that reason, we determined the concentration of soluble thiol compounds of strain Sm777 cells in response to Cd. We noticed an increase of intracellular cysteine pool when bacteria were grown in the presence of 500 µM CdCl_2_ (Fig. 2). Unlike other bacteria or yeast, no modification of gluthatione content was observed [21]. Moreover, no modification of the intracellular pool of cysteine was observed in response to the following metals: NiSO_4_, CuSO_4_, Pb(NO_3_)_2_, ZnSO_4_, CoCl_2_, HgCl_2_, AgNO_3_, tellurite and selenite. The increase of intracellular pool of cysteine might reduce the bioavailability of Cd.

Park and Imlay [22] have shown that high levels of intracellular cysteine promote oxidative DNA damage by driving the Fenton reaction. They actually found that when cysteine homeostasis is disrupted, intracellular cysteine acts as an adventitious reductant of free iron and thereby promotes oxidative DNA damage.

The toxic effect of Cd is mainly mediated by its high degree of reactivity with S, O and N atoms in biomolecules. Cysteine promotes an oxidative stress in cells, however it also protects against Cd toxicity probably by chelating Cd. The resulting metal thiolate complex formation may neutralize the toxicity of heavy metal. To deal with this dilemma, increasing the intracellular cysteine pool, bacterial cells are potentially exposed to an oxidative stress, but these cysteine residues may be stabilized by formation of Cd-cysteine complex decreasing that way the amount of free Cd and free cysteine.

### Formation of CdS particles

**Figure 3:**
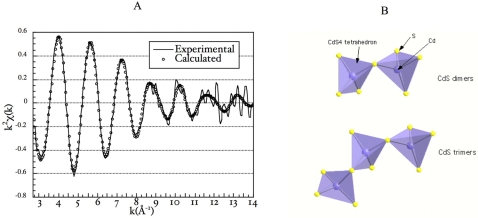
Extended X-ray Absorption Fine Structure (EXAFS) Spectroscopy. (A) Comparison between experimental and calculated EXAFS of *S. maltophilia* Sm777 strain cells. (B) Modeling of Cd dimers and trimers.

When strain Sm777 was grown under aerobic conditions on solid media containing 500 µM CdCl_2_, it formed yellow colonies (Fig. 1C). This observation suggested that bacterial cells may have transformed the Cd(II) into CdS as previously reported for *Klebsiella pneumoniae* [23], and for *Klebsiella planticola* [24]. To test this hypothesis, we used Cd K-edge EXAFS spectroscopy to probe the detailed coordination environment of the metal. The EXAFS spectrum was adjusted using different atomic neighbors around Cd. The nature, number and distances of atoms surrounding Cd in the sample are detailed in Table 1 and the calculated and experimental EXAFS curves are compared in Fig. 3A. EXAFS modeling indicated that the first coordination sphere of Cd was composed of four sulfur atoms at 2.50 and 2.64 Å and confirmed the formation of CdS compounds. EXAFS calculations also indicated the presence of Cd in the second coordination sphere at 3.42 and 3.68 Å. These Cd-Cd contributions indicated that CdS_4_ tetrahedra present in the cell bond to form Cd-S-Cd clusters. The low number of Cd atoms around each Cd (1.3) suggested that the size of the Cd-S-Cd clusters is small and can be a mixture of Cd dimers and trimers as illustrated in Fig. 3B. Thus, Cd-S clusters are formed in the cells and the low coordination number for the Cd-Cd contributions suggests that the product is less crystalline than the CdS reference compound. However, it is not possible to conclude whether these Cd-S clusters are surrounded by poly-thiols molecules or not.

**Table 1:** Cd atomic environment.

Atomic pair	Interatomic distance R (Å)	Debye-Waller parameter (Å)	Number of atoms	Residue
Cd↔S	2.50	0.090	3.1	0.02
Cd↔S	2.64	0.100	0.9	
Cd↔Cd	3.42	0.092	0.4	
Cd↔Cd	3.68	0.110	0.9	

Structural parameters of the Cd atomic environment derived from EXAFS modeling of the Cd K edge EXAFS spectrum of *S. maltophilia* Sm777 bacterial cells.

The mechanism underlying the formation of CdS by strain Sm777 remains unclear; it is obvious that strain Sm777 formed CdS under aerobic conditions, whereas the formation of CdS in *Clostridium thermoaceticum* is mediated by the production of H_2_S under stringent reductive conditions [25]. The aerobic sulfide production and Cd precipitation by *Escherichia coli* was possible by over-expression of the *Treponema denticola* cysteine desulfhydrase gene which product converts cysteine to sulfide under aerobic conditions. However, Cd precipitation as CdS was effective only when cysteine was added to the growth medium [26], whereas the production of CdS by strain Sm777 did not require any exogenous supply of cysteine. The high increase of intracellular pool of cysteine suggests that the bacterium reorients its metabolism to the production of cysteine that might be converted to sulfide used for CdS formation. Cysteine is able to form high-affinity metal ligand clusters and to promote the formation of CdS particles.

Alonso and colleagues [20] showed that a *Stenotrophomonas* strain has acquired a cluster of antibiotic and heavy metal resistance genes from Gram positive bacteria. Most of these genes are homologues of genes previously found on *Staphylococcus aureus* plasmids. In the present study, we evidenced the high tolerance to various heavy metals by *S. maltophilia* Sm777. To our knowledge, this is the first report indicating the high ability of a member of this species to tolerate and to detoxify several heavy metals. This bacterial species is also described as an opportunistic pathogen responsible for nosocomial infections. The severity of these infections is due to the virulence factors of the bacteria and to their occurrence in debilitated patients in whom invasive devices are used. To get more insight in the different mechanisms of heavy metals tolerance, and to identify pathogenesis related genes, it would be of great interest to perform a genome analysis and functional genomic studies of this species.

## Materials and Methods

### Growth conditions

*S. maltophilia* Sm777 was grown aerobically in an incubating shaker at 30°C in tenfold diluted tryptic soy broth (TSB/10) (DIFCO Laboratories, Detroit, USA). For growth on plates, media were solidified with 15 g.l^−1^ Bacto-agar (DIFCO Laboratories, Detroit, USA).

### Determination of metals and antibiotics maximum tolerance concentrations

To determine the MTCs (maximal tolerated concentration) for different heavy metals, bacteria were grown on 10 ml of TSB/10 in the presence of different concentrations of different metals, CdCl_2_, NiSO_4_, CuSO_4_, Pb(NO_3_)_2_, ZnSO_4_, CoCl_2_, HgCl_2_, uranyl acetate and AgNO_3_, at 30°C under shaking. The MTCs corresponded to the highest concentration of each metal at which growth was still observed [27]. The MTCs for the four antibiotics, kanamycin, gentamycin, nalidixic acid, and tetracycline were also determined, and are expressed in µg.ml^−1^. Experiments were performed in triplicate for each condition.

### Analysis of cadmium accumulation

To determine the Cd content of bacterial cells grown in TSB/10 supplemented with 500 µM CdCl_2 _for 48 h cells were harvested, rinsed three times using TSB/10 and dried at 55°C for 24 h . Following addition of 5 ml HNO_3_ (70%), mineralization was carried out in a microwave oven (Mars X; CEM Corp., Matthews, N.C.). Metal content was determined using an inductively coupled plasma atomic emission spectrometry (ICP-OES device; Varian); standard solutions were supplied by Merck.

### Soluble thiols analysis

Cells were harvested and rinsed with TSB/10 and stored at −80°C until analysis. Nonprotein thiols were extracted by disruption of cells by sonication of 5 to 7 mg of frozen bacteria in 0.5 to 0.7 ml of extraction buffer (6.3 mM diethylenetriamine pentaacetic acid [DTPA]−0.1% [vol/vol] trifluoroacetic acid). Thirty microliters of 100 µM *N*-acetyl-L-cysteine was added as an internal standard. The homogenate was centrifuged at 10,000× *g* for 15 min at 4°C (Centromix 1236 V, Rotor 20RT) and the supernatant was filtered (0.22 µm). The derivatization procedure was modified from Rijstenbil and Wijnholds [28]. Filtered extracts (125 µl) were mixed with 225 µl of reaction buffer [0.2 M 4-(2-hydroxy-ethyl)-piperazine-1-propanesulfonic acid pH 8.2 containing 6.3 mM DTPA] and 5 µl of 25 mM monobromobimane dissolved in acetonitrile. Following 15 min of incubation in the dark at room temperature, the reaction was stopped by adding 150 µl of 1 M methane sulfonic acid. The samples were stored at 4°C in the dark until high-performance liquid chromatography (HPLC) analysis. The bimane derivatives were separated on a reversed-phase Nova-Pak C_18_ analytical column (pore size, 60 Å; particle size, 4 µm; dimensions, 3.9 by 300 mm; Waters catalog no. 11695) using two eluents (0.1% [vol/vol] trifluoroacetic acid in water and acetonitrile) at a flow rate of 1 ml.min^−1^. Fluorescence was monitored by a Waters 464 detector (λ_excitation_ = 380 nm; λ_emission_ = 470 nm). Calibration curves of glutathione were used in all measurements. Cysteine, GSH and -glutamylcysteine (-EC) (from Sigma) were used as standard.

### ESEM-EDX and TEM-EDX observations

For transmission electron microscopy (TEM), bacterial cells were harvested from TSA/10 plates containing tellurite (1 mM) or selenite (10 mM). Cells were then fixed in 2.5% glutaraldehyde and postfixed with osmium tetroxide in sodium cacodylate buffer. Dehydration was performed in ethanol and inclusion in epoxy resin. Ultrathin sections were made using a Reichert ultramicrotome. Electron micrographs and chemical microanalyses were obtained with a Jeol (Tokyo, Japan) 100CX transmission electron microscope coupled with an energy dispersive X-ray spectrometer (EDX). Environmental scanning electron microscopy (ESEM) microscope coupled with an energy dispersive X-ray spectrometer (EDX) observations were realized on colonies grown on TSA/10 containing 500 µM CdCl_2_.

### Extended X-ray Absorption Fine Structure (EXAFS) Spectroscopy

Cd K-edge XAS experiments were carried out at the European Synchrotron Radiation Facility (ESRF, Grenoble-France) on the FAME (BM30-b) beamline with Si (220) monochromator crystals using the fluorescence detection mode. The storage ring was operated at 6 GeV with a current of 200 mA. XAS spectra were scanned from 100 eV below to 800 eV above the Cd K-edge. The pre-edge part was extracted from the XANES (X-ray Absorption Near Edge Structure) region (extended from 26600 eV to 26650 eV). XANES spectra intensity was normalized by fitting the photoelectric background above the absorption edge with a 2^nd^ order polynomial function. The EXAFS (Extended X-ray Absorption Fine Structures) data reduction was done using a series of programs developed by Michalowicz [29] based on standard procedures [30]. The extracted EXAFS was k^2^ weighted (with k = wave vector) to enhance the high-k region and Fourier transformed over the k range 2.4 to 14–15 Å^−1^, to R space using a kaiser apodization window with t = 2.5. The resulting pseudo-Radial Distribution Functions (RDF) are uncorrected for phase shift leading to a shift of the peaks by 0.3–0.4 Å. Separate peaks in the RDF corresponding to successive shells of neighboring atoms around Cd were isolated by Back-Fourier Transformation (BFT) for single or multiple shell analysis. The analysis of partial c(k) was based upon the curved wave EXAFS formalism [31] in the single scattering approximation. Curve fitting was performed with a non linear least-square procedure, and phase (f_backscatterer_(k), d_central atom_(k) ) and amplitude (|f_backscatterer_(q, k, R)|) functions used were calculated with FEFF8 32]. Phase and amplitude functions of Cd-S and Cd-Cd atomic pairs were tested on reference compounds (Cd(OH)_2_, CdS).
